# One Anastomosis Gastric Bypass in 6722 Patients: Early Outcomes from a Private Hospital Registry

**DOI:** 10.3390/jcm12216872

**Published:** 2023-10-31

**Authors:** Nasser Sakran, Shiri Sherf-Dagan, Keren Hod, Uri Kaplan, Bella Azaria, Asnat Raziel, Assuta Bariatric Surgeons Collaborative

**Affiliations:** 1 Assuta Medical Center, Tel Aviv 6971028, Israel; shiris@ariel.ac.il (S.S.-D.); hodkeren@gmail.com (K.H.); kaplanuri@gmail.com (U.K.); bellaa@assuta.co.il (B.A.); doctor@asnatraziel.com (A.R.); 2 Department of General Surgery, Holy Family Hospital, Nazareth 1601001, Israel; 3 The Azrieli Faculty of Medicine Safed, Bar-Ilan University, Ramat Gan 5290002, Israel; 4 Department of Nutrition Sciences, School of Health Sciences, Ariel University, Ariel 4077625, Israel; 5 Department of Surgery, Emek Medical Center, Afula 1812601, Israel; 6 Rappaport Faculty of Medicine, Technion—Israel Institute of Technology, Haifa 3200003, Israel

**Keywords:** severe obesity, bariatric surgery, one anastomosis gastric bypass, early postoperative complications

## Abstract

Background: One-anastomosis gastric bypass (OAGB) is an emerging metabolic bariatric surgery (MBS) type used in both primary OAGB (pOAGB) and revisional OAGB (rOAGB). We studied ≤30-day outcomes of pOAGB and rOAGB and identified predictors of early complications. Methods: Electronic medical records of all OAGBs performed between January 2017 and December 2021 at a high-volume bariatric clinic in Israel comprising four hospital centers were scanned retrospectively using specialized data software (MDClone software, version 6.1). Data gathered were patients’ characteristics, surgical procedure, and ≤30-day complications with Clavien–Dindo Classification (CDC). Multivariate logistic regression analyses were used to identify factors related to early complications of pOAGB and rOAGB. Results: A total of 6722 patients underwent a pOAGB (*n* = 5088, 75.7%) or rOAGB (*n* = 1634, 24.3%) procedure at our institution. Preoperative mean age and body mass index (BMI) were 40.6 ± 11.5 years and 41.2 ± 4.6 kg/m^2^, respectively. Early complications occurred in 258 (3.8%) patients (176 pOAGB and 82 rOAGB) and included mainly bleeding (*n* = 133, 2.0%), leaks (*n* = 31, 0.5%), and obstruction/strictures (*n* = 19, 0.3%). CDC complications for grades 1–2 and grades 3a-–5 were 1.5% and 1.6%, respectively. The overall mortality rate was 0.03% (*n* = 2). Age, operative time ≥3 h, and any additional concomitant procedure were independent predictors of early complications following pOAGB, while a diagnosis of diabetes mellitus and operative time ≥3 h were independent predictors of early complications following rOAGB. Conclusions: OAGB was found to be a safe primary and revisional MBS procedure in the ≤30-postoperative day term. The most common complications were gastrointestinal bleeding, leaks, and obstruction/stricture.

## 1. Introduction

Obesity is a prevalent chronic disease associated with an increased risk of coexisting medical conditions and premature mortality [1,2,3]. Metabolic bariatric surgery (MBS) is presently the most effective and durable treatment for patients with clinically severe obesity [4,5,6].

The number of MBS procedures has increased over the last decades [7]. One anastomosis gastric bypass (OAGB), a modification of the original Mason loop gastric bypass, was first reported by Rutledge in 1997 [8]. Over the past two decades, OAGB has gained widespread acceptance and, in recent years, has been endorsed as an acceptable MBS procedure with an acceptable complication rate by both the American Society for Metabolic and Bariatric Surgery (ASMBS) and the International Federation for the Surgery of Obesity and Metabolic Disorders (IFSO) [9]. OAGB consists of a long, narrow sleeve gastric pouch in conjunction with end-to-side or side-to-side gastrojejunostomy performed 150–200 cm distal to the ligament of Treitz [10].

The popularity of OAGB has gradually risen worldwide, and currently, it ranks as the third most common MBS [11]. In Israel, OAGB has emerged as the most frequently performed MBS procedure in recent years, both as a primary and revisional operation [12]. Contributing factors to its rise likely include shorter operative duration and the failure of former restrictive MBS procedures [10,11].

OAGB has been found to be highly effective in terms of weight loss, improvement of comorbidities, and quality of life [12,13]. Nonetheless, OAGB is considered by some to be a controversial procedure mainly due to the theoretical risk of short- and long-term complications, including the consequences of chronic bile reflux, a higher rate of marginal ulcers, and a lack of long-term nutritional data [14,15].

Outcomes of several short- and mid-term OAGB patient series have been published by teams from the USA, Germany, Italy, Spain, India, Taiwan, Iran, Egypt, France, the UK, and Israel, but are often in cohorts with small sample sizes [16,17,18,19,20,21,22,23,24].

This study aimed to evaluate the ≤30-day postoperative safety of primary OAGB (pOAGB) and revisional OAGB (rOAGB) based on a large electronic medical records database at a high-volume bariatric center in Israel. Other goals were to identify factors that might predict 30-day complications following OAGB and report the causes and management of potentially difficult complications that can occur within the ≤30-day postoperative period.

## 2. Materials and Methods

### 2.1. Study Design

A retrospective cohort study analyzed an electronic database recording all pre-, peri-, and early postoperative data of all patients who underwent MBS at Assuta Bariatric Centers (ABC, Assuta, Israel). ABC is part of Assuta Medical Centers, which is the largest private hospital chain in Israel. The database was stored in MDClone (ADAMS Healthcare Data Platform, Beer Sheva, Israel, version 6.1). This study received approval from the Assuta Medical Centers’ Institutional Review Board ethics committee (approval number 43-20-ASMC). Informed consent was waived due to the retrospective and anonymous nature of data collection.

### 2.2. Patient Inclusion and Exclusion

Patients were included if they were 18 years of age or older, had undergone either pOAGB or rOAGB procedures at one of the four ABCs, and had completed a 30-day post-operative follow-up. All patients obtained approval for MBS from our medical centers’ multidisciplinary bariatric committee [25].

Routinely, all patients who undergo MBS at ABC are monitored by phone calls at 1, 7, 30 days, and 3, 6, 9, 12, and 24 months post-discharge from the hospital. Moreover, patients are instructed to attend the center where the operation was performed in case of a complication during the first 30 days after surgery.

### 2.3. Outcome Measures

Demographic and clinical characteristics, operative time, length of stay (LOS), and ≤30-day complications, readmission, reoperation, and mortality rates were assessed. The Clavien–Dindo Classification (CDC) system [26] was used to rank perioperative complications by their level of morbidity and the therapy used to treat them.

### 2.4. Surgical Technique

OAGB was performed in patients following an extensive preoperative workup according to pre-defined criteria [27]. The OAGB is typically performed laparoscopically using five trocars. A small window is made in the lesser omentum just above the lesser curvature of the stomach, entering the lesser sac 2 cm below the incisura. The cornerstone of the procedure is the creation of a long and narrow gastric tube based on the lesser curvature. The use of a 34–36 Fr bougie in creating the tube is important for the restriction of gastric volume. The stomach is divided obliquely along a line extending from the incisura to the angle of His alongside a 34–36 Fr bougie to maintain an approximately 1 cm pouch diameter. No short gastric vessels are divided.

An enterotomy is created on the small bowel, approximately 150–200 cm distal to the ligament of Treitz. A gastrojejunostomy is performed using a linear stapler to anastomose the gastric pouch to the small bowel. The gastrojejunal enterotomy is closed with a one-layer, full-thickness, absorbable running suture. The afferent limb is placed on the patient’s left side and the efferent limb on the patient’s right side to avoid torsion of the intestinal mesentery. An intraoperative methylene blue test is performed to check for leaks.

### 2.5. Postoperative Care

No routine upper gastrointestinal (UGI) imaging series is performed. Oral intake is restarted on the first postoperative day, and usually, patients are discharged on the second postoperative day with specific dietary instructions. To prevent deep venous thrombosis, patients receive a daily subcutaneous injection with low-molecular-weight heparin for ≤10 days postoperatively. A proton pump inhibitor is administered routinely for up to 6 months. Except in unusual instances, a non-opioid regimen was used for pain control.

### 2.6. Statistical Analysis

Analyses were performed using SPSS version 28.0. All analyses are presented for the entire study population and for the pOAGB and rOAGB groups separately. Continuous variables were reported as mean and standard deviation (SD) and/or median and interquartile range (IQR), and categorical variables as frequencies and percentages. The Cuzick’s test was used to analyze the trend in case volume for pOAGB and rOAGB. Univariable analyses (i.e., independent t-test, Mann–Whitney U test, or Chi-square test) were used to compare demographics and clinical outcomes between the pOAGB and rOAGB groups. Multivariable logistic regression models using the forward stepwise selection method were applied to identify risk factors related to early postoperative complications after either pOAGB or rOAGB procedures.

Independent variables inserted into these models are those with a significant association with complications (*p* < 0.1), as found by a previous univariate analysis, or those with potential clinical significance. Independent variables that were highly correlated (r ≥ 0.75) were not included in these multivariable logistic regression models simultaneously, even if they were significantly associated with complications, in order to avoid multicollinearity. Sub-analyses were performed to compare rOAGB patients that underwent laparoscopic adjustable gastric banding (LAGB), sleeve gastrectomy (SG), and silastic ring vertical gastroplasty (SRVG) in the past. These sub-analyses included only rOAGB patients that had one previous MBS procedure (i.e., patients with multiple [≥2] MBS procedures in the past were excluded from this analysis). The comparisons were conducted using one-way ANOVA, Kruskall–Wallis, or chi-square tests, as appropriate. All statistical tests were two-tailed, and statistical significance was set at *p* < 0.05.

## 3. Results

### 3.1. Patient and Surgical Characteristics

**Figure 1:**
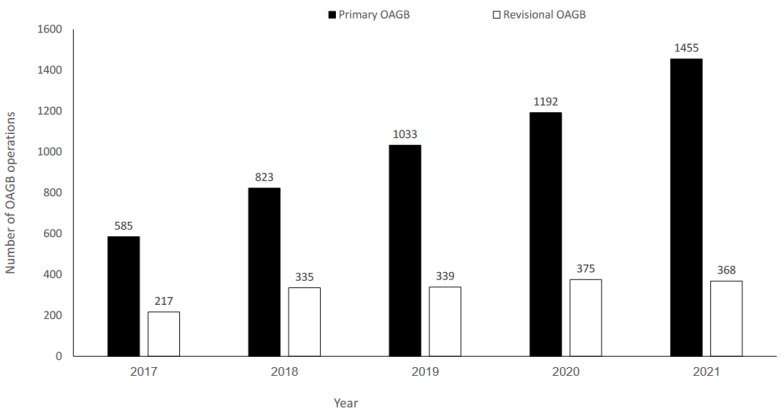
Number of primary and revisional one-anastomosis gastric bypass (OAGB) operations during 2017–2021.

A total of 6722 patients who underwent OAGB between January 2017 and December 2021 were identified; 5088 (75.7%) had pOAGB, and 1634 (24.3%) had rOAGB. There was a significant increase in pOAGB volume over time (*p* = 0.002); however, rOAGB volume remained stable (Figure 1).

Patients’ preoperative demographic and clinical characteristics are presented in Table 1. The mean ± SD of preoperative age and BMI were 40.6 ± 11.5 years and 41.2 ± 4.6 kg/m^2^, respectively, and 75.1% of patients were females. The pOAGB group was significantly younger than the rOAGB group (39.6 ± 11.7 versus 43.8 ± 10.4 years, *p* < 0.001). Moreover, the prevalence of preoperative gastroesophageal reflux disease (GERD) was significantly lower in the pOAGB group compared to the rOAGB group (14.4% versus 23.3%, *p* < 0.001, respectively).

Surgical characteristics are presented in Table 2. Operative time and hospitalization length of stay were significantly longer among the rOAGB group compared to the pOAGB group (81.5 ± 33.9 versus 62.7 ± 21.9 min and 2.4 ± 2.3 days versus 2.2 ± 0.9 days, *p* < 0.001, respectively). The laparoscopic approach was used in 6714 (99.9%) cases. One case was performed using an open approach due to a large ventral hernia that was repaired during revisional OAGB, and 7 cases (2 pOAGB and 5 rOAGB cases) were converted to open surgery because of bleeding (*n* = 3), obstruction (*n* = 3), and colon injury (*n* = 2).

Previous bariatric procedures in the rOAGB group (*n* = 1634) included LAGB (*n* = 1074 [65.7%]), SG (*n* = 423 [25.9%]), silastic ring vertical gastroplasty (SRVG) (*n* = 69 [4.2%]), and different combinations of these three surgery types (*n* = 68 [4.2%]). The incidence of prior non-bariatric abdominal surgery was significantly higher in the rOAGB group (*n* = 157 [9.6%]) compared to the pOAGB group (*n* = 237 [4.7%]), *p* < 0.001.

### 3.2. Early Complications

Early complications (i.e., ≤30 days) occurred in 258 (3.8%) patients, 176 (3.5%) within the pOAGB group, and 82 (5.0%, *p* = 0.006) within the rOAGB group (Table 2). The most common early complication was gastrointestinal (GI) bleeding in 133 patients (2.0%), with no significant difference between the pOAGB and rOAGB groups. Of these patients, intraluminal bleeding was treated conservatively in 59.4% of cases, endoscopically in 26.6%, and 14.1% of cases necessitated reoperation. Extraluminal bleeding was treated conservatively in 66.2% of cases; 33.8% necessitated reoperation. Leaks occurred in 31 patients (0.5%) with no significant difference between the pOAGB and rOAGB groups. The most common location was the gastroentero-anastomosis (GEA) (68.8%), followed by the staple line (15.6%) and small bowel (15.6%). Conservative treatment was applied in 20.0% of all leak events. Operative treatment was performed in 77.2% of anastomotic leaks, 60.0% of staple-line leaks, and 80% of small bowel injuries. Obstruction/stricture occurred in 19 (0.3%) patients (10 [0.6%] within the rOAGB group versus 9 [0.2%] in the pOAGB group, *p* = 0.012]). Endoscopic treatment was successful in 33.0% of cases; operative treatment was required in 66.0% of patients. The most common sites for obstruction were the GEA, occurring in 9/19 cases (47.4%), early adhesions in 6 cases (31.6%), and port site hernias in 3 cases (15.8%). Sub-analyses assessing the postoperative risk of complications based on specific prior primary MBS procedures were performed. Proportionally, results indicated that prior SRVG had statistically significantly more leak (*p* = 0.037) and respiratory (*p* = 0.007) complications and resulted in a higher rate of readmissions (*p* = 0.001) (Table 3).

Table 4 summarizes the distribution of complication severity. Bleeding was the most common cause of minor (*n* = 84, 80.8%) and major (*n* = 49, 44.9%) complications in both pOAGB (*n* = 62 [80.5%] and *n* = 35 [55.5%]) and rOAGB groups (*n* = 22 [81.5%] and *n* = 14 [30.4%]). The percentages of total pOAGB and rOAGB patients with complications within CDC categories are presented in Supplementary Figure S1. The incidence of major complications (CDC 3a-5) was significantly higher in the rOAGB group compared to the pOAGB group (46 [2.8%] vs. 63 [1.2%], *p* = 0.002, respectively) (Table 4).

**Figure 2:**
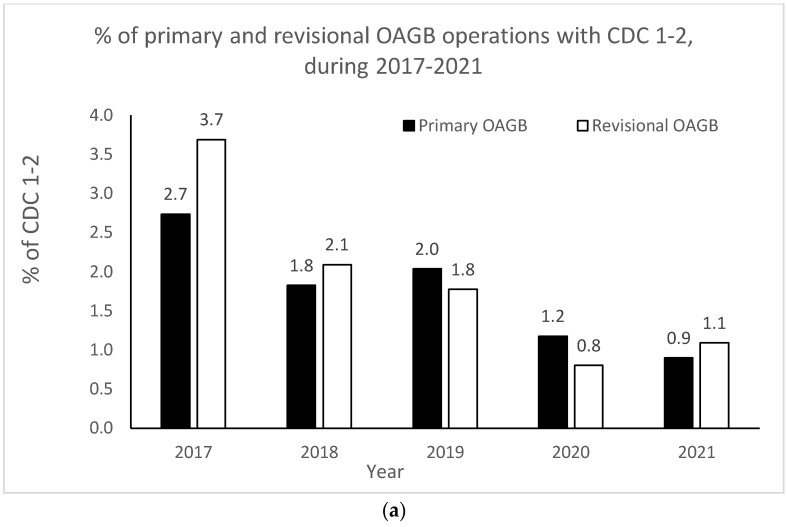

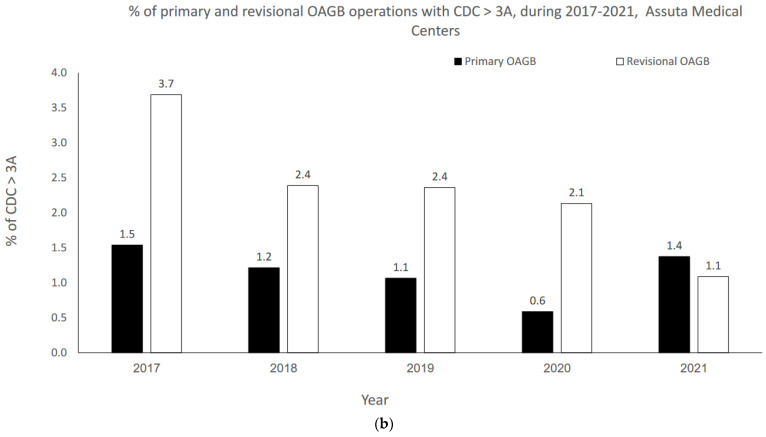
(**a**) Percent of minor primary and revisional one-anastomosis gastric bypass (OAGB) complications (Clavien–Dindo classification [CDC] 1 to 2) in relation to the number and type of procedures per year (2017–2021). (**b**). Percent of major primary and revisional one-anastomosis gastric bypass (OAGB) complications (Clavien–Dindo classification [CDC] 3a to 5) in relation to number and type of procedures per year (2017–2021).

The OAGB learning curve in our center with respect to CDC categories is graphically portrayed in Figure 2a,b. Minor complication (i.e., CDC 1–2) rates significantly decreased as the number of procedures increased over time (r = −0.900, *p* = 0.037 for both pOAGB and rOAGB). Major complication (i.e., CDC > 3a-5) rates significantly decreased as the number of procedures increased over time only for rOAGB procedures (r = −0.975, *p* = 0.005), while for pOAGB procedures a downward trend was observed (r = −0.400, *p* = 0.505). Our centers’ 2017 rates of CDC complication categories 1–2 (Figure 2a) were 2.7% and 3.7% for pOAGB and rOAGB procedures, respectively; by 2021, the rates had fallen to 0.9% and 1.1%, probably due to a 127.5% increase in the number of OAGB cases performed.

Potential predictors of early complications are presented in Table 5 (pOAGB) and Table 6 (rOAGB). The variables inserted into the multivariate logistic model of pOAGB were those with significant association (*p* < 0.1) with early postoperative complications, as shown in Table 5 (i.e., age [mean ± SD], operative length ≥ 3 h, any additional concomitant procedure, and aspirin treatment) and those with potential clinical significance (i.e., sex, baseline BMI ≥ 50 kg/m^2^, diagnosis of hypertension or diabetes mellitus).

Following a forward stepwise selection method, the final multivariate logistic model showed that age (OR = 1.02, 1.00–1.03; *p* = 0.023), operative time ≥ 3 h (OR = 5.04, 1.08–23.62; *p* = 0.040), and any additional concomitant procedure (OR = 1.63, 1.11–2.38; *p* = 0.012) were independent predictors of early postoperative complications following pOAGB. In a similar fashion, as presented in Table 6, the variables inserted into the multivariate logistic model for prediction of early postoperative complications in rOAGB patients were: age (mean ± SD), operative time ≥ 3 h, additional partial gastrectomy or ventral hernia repair, previous vertical banded gastroplasty, as well as the above-listed variables with potential clinical significance (i.e., sex, baseline BMI ≥ 50 kg/m^2^, and a diagnosis of hypertension or diabetes mellitus). Following a stepwise procedure, the final multivariable logistic model showed that a diagnosis of diabetes mellitus (OR = 1.81, 95% CI 1.01–3.23; *p* = 0.047) and operative time ≥ 3 h (OR = 4.74, 95% CI 1.56–14.39; *p* = 0.006) were independent predictors of early postoperative complications following rOAGB.

### 3.3. Early Reoperations and Mortality

Early reoperation was required in 61 OAGB patients (0.9%), 39 (0.8%) pOAGB, and 22 (1.3%) rOAGB (*p* = 0.036) (Table 2); a single reoperation was performed in 56 patients, 4 patients required 2 reoperations, and one patient required 4 reoperations. In this patient, two operations were performed due to a leak at the area of the anastomosis. In the third operation, the patient was converted to Roux-en-Y gastric bypass (RYGB), and the last operation was performed to address drainage of an intra-abdominal abscess. Of reoperated patients, the two most common reasons for reoperation were bleeding [26 (42.6%)] and leakage [22 (36%)].

The early mortality rate was 0.03% (*n* = 2). The first patient, a 56-year-old female, died in 2017 after rOAGB for a previous SG. Her medical history included fatty liver disease and a previous open total abdominal hysterectomy. Her preoperative BMI was 49.7 kg/m^2^. On postoperative day 1, the patient developed severe septic shock. Computed tomography (CT) of the abdomen revealed a leak from the GEA. She was taken to the operating room and, during induction, developed cardiac arrest. Resuscitation and CPR were unsuccessful.

The second patient, a 64-year-old female, died in 2020 after a pOAGB procedure. Her preoperative BMI was 46.9 kg/m^2^, and her past medical history included diabetes mellitus, hyperlipidemia, and fatty liver disease. The patient developed bleeding at the GEA on postoperative day 1. She underwent successful endoscopic treatment and was discharged two days later. One week after her discharge, she presented to the emergency department in severe septic shock. An extremely large amount of free air was found on the CT scan, and the patient was transferred to the operating room. Laparotomy revealed a leak at the GEA, which was repaired primarily. Over the course of the next 24 h, the patient’s condition continued to deteriorate. Despite close observation and intensive medical measures taken, the patient died on the first postoperative day.

## 4. Discussion

OAGB is currently the third most performed MBS procedure worldwide after SG and RYGB [27] and has become the most common MBS procedure in Israel in recent years [12]. To the best of our knowledge, the current study provides outcomes for the largest series of consecutive patients (*n* = 6722) who underwent OAGB as a primary or revisional procedure. 

### 4.1. Operating Time and Length of Stay

The mean operating time in the current study was 67.3 min, within the range of the recently published systematic review of 22 studies (*n* = 12,807), which found mean operative times ranging from 35–157 min [28]. Our patients’ median length of hospital stay was 2 days for both primary and revisional OAGB patients, well within the reported range of 1 to 5 days for most post-OAGB patients [8]. A prolonged stay is seen in 2.0% to 3.0% of patients and may be longer (4–11 days) [29] during the early learning curve, in patients with a higher BMI, or in those undergoing rOAGB [30,31,32]. 

### 4.2. Early Complications

Early postoperative complication rates reported in the literature following OAGB vary from 3.5% to 7.5% [33]. Common complications include leaks, intra-abdominal bleeding, suture-line hemorrhage, stricture, and major organ injury. Major complications after OAGB, defined by the need for transfusion, return to the operating room, and/or prolonged length of hospital stay, are seen in 2.0% to 3.0% of patients [29].

Generally, intraluminal and extraluminal leaks and bleeding are noted primarily within the first three days following surgery, and almost all are detected within the first 30 days [16,34]. The early postoperative bleeding after OAGB is most likely related to staple-line problems, but it may also arise from the short gastric vessels or spleen during dissection. The most common early complication in the present series (as in other OAGB series [28]) was GI bleeding in 2.0% of patients, with no significant difference between pOAGB and rOAGB patients. A minority of cases of bleeding generally require surgical intervention [18,35]. The majority of postoperative bleeds in the present series were managed conservatively (>60.0%). Early recognition and care of intraoperative and postoperative bleeding are crucial.

The leakage rate in our series was 0.5% (0.4% pOAGB, 0.7% rOAGB; NS), consistent with that reported in our previous series (0.5%, *n* = 1/182) [16] and lower than reported in a recent systematic review (0.96%) [28]. In most cases, operative treatment was required. Leaks after OAGB can result in severe peritonitis, sepsis, and multi-organ failure. After OAGB, leaks most commonly result at the staple line of the gastric pouch, gastric remnant, or GJ anastomosis and occur in 0.7% to 2.0% of cases [36,37]. The rate of 30-day leak in the 11-center Longitudinal Assessment of Bariatric Surgery (LABS) consortium study of consecutive patient outcomes ranged from 0.6 to 4.4% in MBS procedures. The technical factors associated with leaks in this group were revisional surgery (*p* < 0.001), use of an abdominal drain (*p* = 0.02), and open surgery (*p* < 0.001) [38]. The rates of both primary (0.4%) and revisional (0.7%) OAGB leaks in the current series of 6722 patients are at the low end of the range of LABS 30-day leak outcomes.

Stenosis can occur if the anastomosis narrows, kinks, or twists, while symptoms usually begin within the first six weeks after surgery. In the current study, stenosis at the GEA was encountered in 9/19 patients with obstruction. All were readmitted because of vomiting due to a narrowing of the anastomosis. A minority of cases (33.0%) required endoscopic intervention, and in the majority of patients (66.0%), an operative approach was chosen. The practice in ABC was to perform a gastrograffin swallow test on postoperative day 1. Most patients had minimal or no passage of contrast material through the GEA. Due to the early presentation of stenosis, some of the surgeons chose to perform an early revision of the anastomosis instead of an endoscopic treatment. Although stenosis/strictures are uncommon, their early recognition is important as they require urgent surgery to be remedied [39].

Interestingly, in subgroup analysis, results suggested that prior SRVG may be associated with a greater risk of readmission and revision following rOAGB because its rates of leak and respiratory complications were proportionally higher than in patients with prior LAGB and SG; however, sample sizes were too small to draw definitive conclusions. In terms of our centers’ learning curve, in the current study, as the number of procedures increased annually, a significant decrease in minor complication rates was seen. The mortality rate in our series was 0.03% (2 of 6722 patients), among the lowest reported incidences following OAGB in the literature [25,31].

### 4.3. Limitations and Strengths

This study’s retrospective design is a limitation, although the very large sample size is a counterbalancing strength. In the present study, phone call monitoring at 30-day postoperatively was available for 76% of the cohort, but as patients are instructed to attend the surgical center in case of any complications during the first 30-day postoperatively, we assume that the missing information for that period was negligible. Future comparative and randomized studies will elucidate more clearly the specific advantages and disadvantages of OAGB relative to other MBS procedures. Future studies with longer follow-ups are needed to investigate late postoperative complications following OAGB.

## 5. Conclusions

In this retrospective cohort of 6722 patients, OAGB operating time was relatively short, with a similarly low <30-day complication rate in both primary and revisional OAGB procedures and a relatively low mortality rate at a high-volume bariatric center. Age, ≥3 h of operating time, and any additional concomitant procedure with OAGB were independent predictors of early complications following pOAGB, while ≥3 h of operating time and a diagnosis of diabetes mellitus were independent predictors of early complications following rOAGB.

## Figures and Tables

**Table 1:** Patient preoperative demographic and clinical characteristics.

Variable	Total Study Population (*n* = 6722)	pOAGB Group (*n* = 5088)	rOAGB Group (*n* = 1634)	*p* Value *
Age (years), mean ± SD	40.6 ± 11.5	39.6 ± 11.7	43.8 ± 10.4	<0.001
Female, *n* (%)	5045 (75.1)	3814 (75.0)	1231 (75.3)	0.768
BMI (kg/m2), mean ± SD	41.2 ± 4.6	41.2 ± 4.5	41.2 ± 4.8	0.708
Diabetes mellitus, *n* (%)	1847 (27.5)	1401 (27.5)	446 (27.3)	0.873
Hypertension, *n* (%)	1481 (22.0)	1071 (21.0)	410 (25.1)	<0.001
Hyperlipidemia, *n* (%)	2501 (37.2)	1904 (37.4)	597 (36.5)	0.537
Obstructive sleep apnea, *n* (%)	532 (7.9)	411 (8.1)	121 (7.4)	0.400
Fatty liver disease (NAFLD/NASH), *n* (%)	4646 (69.1)	3606 (70.9)	1040 (63.6)	<0.001
Gastroesophageal reflux disease, *n* (%)	1112 (16.5)	731 (14.4)	381 (23.3)	<0.001

BMI, body mass index; NAFLD, nonalcoholic fatty liver disease; NASH, non-alcoholic steatohepatitis. * *p*-value between primary (pOAGB) and revisional (rOAGB) procedures.

**Table 2:** Surgical characteristics and early postoperative complications (≤30-days).

Variable	Total Study Population (*n* = 6722)	pOAGB Group (*n* = 5088)	rOAGB Group (*n* = 1634)	*p* Value *
Operative time (minutes), mean ± SD	67.3 ± 26.6	62.7 ± 21.9	81.5 ± 33.9	<0.001
Length of stay (days), mean ± SD (median)	2.2 ± 1.4 (2.0)	2.2 ± 0.9 (2.0)	2.4 ± 2.3 (2.0)	<0.001
Laparoscopic approach, *n* (%)	6714 (99.9)	5086 (99.9)	1628 (99.6)	>0.999
Patients with previous abdominal surgery, *n* (%) ^†^	394 (5.9)	237 (4.7)	157 (9.6)	<0.001
Patients with concomitant additional procedures, *n* (%)	2122 (31.4)	898 (17.6)	1214 (74.3)	<0.001
Total additional procedures, n	2470 ^††^	942 ^††^	1528 ^††^	
Gastric band removal, n/N (%)	945/2470 (38.3)	0/942 (0.0)	945/1528 (61.8)	<0.001
Laparoscopic cholecystectomy, n/N (%)	811/2470 (32.8)	591/942 (62.7)	220/1528 (14.4)	0.029
Hiatal hernia repair, n/N (%)	534/2470 (21.6)	300/942 (31.8)	234/1528 (15.3)	<0.001
Partial gastrectomy, n/N (%)	99/2470 (4.0)	0/942 (0.0)	99/1528 (6.5)	<0.001
Ventral hernia repair, n/N (%)	81/2470 (3.3)	51/942 (5.4)	30/1528 (1.9)	0.325
Total patients with postoperative complications (some patients had >1 complication)	258 (3.8)	176 (3.5)	82 (5.0)	0.006
Bleeding, *n* (%)	133 (2.0)	97 (1.9)	36 (2.2)	0.475
Leak, *n* (%)	31 (0.5)	19 (0.4)	12 (0.7)	0.090
Obstruction/stricture, *n* (%)	19 (0.3)	9 (0.2)	10 (0.6)	0.012
Infection, *n* (%)	14 (0.2)	6 (0.1)	8 (0.5)	0.009
Respiratory complication, *n* (%)	15 (0.2)	8 (0.2)	7 (0.4)	0.064
Small bowel injury, *n* (%)	3 (0.04)	0 (0.0)	3 (0.2)	0.014
Acute renal failure, *n* (%)	3 (0.04)	1 (0.02)	2 (0.1)	0.148
Readmissions, *n* (%)	130 (1.9)	82 (1.6)	48 (2.9)	0.001
Reoperations, *n* (%)	61 (0.9)	39 (0.8)	22 (1.3)	0.036
Mortality, *n* (%)	2 (0.03)	1 (0.02)	1 (0.06)	0.427

* p-value between primary (pOAGB) and revisional (rOAGB) procedures. ^†^ Previous abdominal surgery includes: cesarean section, cholecystectomy, hernia repair, hysterectomy, appendectomy, and gynecologic surgeries. ^††^ Total additional procedures that were conducted during the OAGB surgery were calculated by a summary of each procedure; therefore, 2122 patients had a total of 2470 concomitant procedures, with multiple patients undergoing more than one procedure.

**Table 3:** Surgical characteristics and early postoperative complications (≤30-days) of rOAGB patients with different prior bariatric procedures.

Variable	Previous LAGB rOAGB Patients (*n* = 1070)	Previous SG rOAGB Patients (*n* = 420)	Previous SRVG rOAGB Patients (*n* = 69)	*p* Value *
Operative time (minutes), mean ± SD	75.3 ± 28.7	88.9 ± 34.6	121.4 ± 55.3	<0.001
Length of stay (days), mean ± SD (median)	2.4 ± 2.1 (2.0)	2.3 ± 1.1 (2.1)	2.8 ± 1.4 (2.5)	<0.001
Laparoscopic approach, *n* (%)	1067 (99.7)	419 (99.7)	68 (98.6)	0.235
Prior abdominal surgery, *n* (%) ^†^	94 (8.8)	35 (8.3)	20 (29.0)	<0.001
Concomitant added procedures, *n* (%)	939 (87.8)	165 (39.3)	54 (78.3)	<0.001
Additional procedures, n	1155 ^††^	211 ^††^	54 ^††^	
Gastric band removal, n/N (%)	889/1155 (76.9)	0/211 (0.0)	0/54 (0.0)	<0.001
Laparoscopic cholecystectomy, n/N (%)	117/1155 (10.1)	72/211 (34.1)	12/54 (22.2)	0.004
Hiatal hernia repair, n/N (%)	118/1155 (10.2)	77/211 (36.5)	19/54 (35.2)	<0.001
Partial gastrectomy, n/N (%)	14/1155 (1.2)	54/211 (25.6)	17/54 (31.5)	<0.001
Ventral hernia repair, n/N (%)	17/1155 (1.5)	8/211 (3.8)	6/54 (11.1)	0.004
Postoperative complications **	53 (5.0)	17 (4.0)	7 (10.1)	0.096
Bleeding, *n* (%)	27 (2.5)	6 (1.4)	0 (0.0)	0.191
Leak, *n* (%)	7 (0.7)	1 (0.2)	2 (2.9)	0.037
Obstruction/stricture, *n* (%)	6 (0.6)	3 (0.7)	1 (1.4)	0.653
Infection, *n* (%)	5 (0.5)	2 (0.5)	0 (0.0)	0.850
Respiratory complication, *n* (%)	4 (0.4)	1 (0.2)	2 (2.9)	0.007
Small bowel injury, *n* (%)	2 (0.2)	1 (0.2)	0 (0.0)	0.914
Acute renal failure, *n* (%)	1 (0.1)	1 (0.2)	0 (0.0)	0.747
Readmissions, *n* (%)	29 (2.7)	10 (2.4)	7 (10.1)	0.001
Reoperations, *n* (%)	15 (1.5)	4 (1.0)	1 (1.4)	0.780
Mortality, *n* (%)	0 (0.0)	1 (0.2)	0 (0.0)	0.257

rOAGB, revisional one-anastomosis gastric bypass; LAGB, laparoscopic adjustable gastric bypass; SG, sleeve gastrectomy; SVRG, silastic ring vertical gastroplasty. * *p*-value between all three rOAGB groups; only rOABG patients with one previous bariatric surgery were included. ** Some patients had >1 complication. ^†^ Previous abdominal surgery includes: cesarean section, cholecystectomy, hernia repair, hysterectomy, appendectomy, and gynecologic surgeries. ^††^ Total additional procedures that were concurrent with the OAGB were calculated by a summary of each procedure; therefore, 939 previous LAGB rOAGB patients underwent a total of 1155 additional procedures during the OAGB surgery.

**Table 4:** Clavien–Dindo classification for grading adverse events.

Complications	Total Study Population (*n* = 6722)	pOAGB Group (*n* = 5088)	rOAGB Group (*n* = 1634)	*p*-Value *
Minor				
CDC grade 1–2, n/N (%)	104/6722 (1.5)	77/5088 (1.5)	27/1634 (1.6)	0.648
Bleeding, n/N (%)	84/6722 (1.2)	62/5088 (1.2)	22/1634 (1.3)	>0.999
Leaks, n/N (%)	5/6722 (0.1)	4/5088 (0.08)	1/1634 (0.06)	>0.999
Obstruction/stricture, n/N (%)	0/6722 (0.0)	0/5088 (0.0)	0/1634 (0.0)	>0.999
Other ^†^, n/N (%)	15/6722 (0.2)	11/5088 (0.2)	4/1634 (0.2)	>0.999
Major				
CDC grade 3a-5, n/N (%)	109/6722 (1.6)	63/5088 (1.2)	46/1634 (2.8)	0.002
Bleeding, n/N (%)	49/6722 (0.7)	35/5088 (0.7)	14/1634 (0.9)	0.056
Leaks, n/N (%)	26/6722 (0.4)	15/5088 (0.3)	11/1634 (0.6)	0.842
Obstruction/stricture, n/N (%)	19/6722 (0.2)	9/5088 (0.2)	10/1634 (0.6)	0.189
Other ^†^, n/N (%)	13/6722 (0.2)	3/5088 (0.06)	10/1634 (0.6)	0.004
Death, n/N (%)	2/6722 (0.03) ^††^	1/5088 (0.01)	1/1634 (0.06)	>0.999

CDC, Clavien–Dindo classification. * *p*-value between primary (pOAGB) and revisional (rOAGB) procedures. ^†^ Others include respiratory complications, superficial and deep infections, and urinary tract infections. One patient had both a respiratory complication and a superficial infection (CDC grade = 4). ^††^ Note: total mortality: 2/6772 (0.03% of the total sample).

**Table 5:** Univariable analysis of factors related to early postoperative complications after primary OAGB performed from 2017–2021 (*n* = 5088).

Factor	Patients without Complications (*n* = 4912)	Patients with Complications * (*n* = 176)	*p*-Value
Female, *n* (%)	3688 (75.1)	126 (71.2)	0.289
Age (years), mean ± SD	39.5 ± 11.6	41.7 ± 12.9	0.027
Age ≥ 60 years, *n* (%)	214 (4.4)	18 (10.2)	0.001
BMI (kg/m^2^), mean ± SD	41.2 ± 4.5	40.8 ± 4.5	0.143
Baseline BMI (kg/m^2^) ≥ 50 kg/m^2^, *n* (%)	220 (4.9)	8 (5.3)	0.847
Smoker, *n* (%)	284 (5.8)	10 (5.7)	>0.999
Operative length (minutes), mean ± SD	62.4 ± 21.4	70.9 ± 32.5	<0.001
Operative length ≥ 3 h, *n* (%)	11 (0.2)	2 (1.2)	0.073
Additional concomitant procedures (%yes) ^†^, *n* (%)	853 (17.4)	45 (25.6)	0.009
Lap. cholecystectomy, *n* (%)	560 (11.4)	31 (17.6)	0.016
Lap. hiatal hernia repair, *n* (%)	289 (5.9)	11 (6.3)	0.746
Lap. ventral hernia repair, *n* (%)	46 (0.9)	5 (2.8)	0.030
Associated medical problems			
Hypertension, *n* (%)	1031 (21.0)	40 (22.7)	0.573
Diabetes mellitus, *n* (%)	1351 (27.5)	50 (28.4)	0.797
Hyperlipidemia, *n* (%)	1833 (37.3)	71 (40.3)	0.428
Obstructive Sleep Apnea, *n* (%)	398 (8.1)	13 (7.4)	0.888
Fatty liver disease, *n* (%)	3491 (71.1)	115 (65.3)	0.109
Aspirin treatment, *n* (%)	199 (4.1)	12 (6.8)	0.081
Hypercoagulopathy, *n* (%)	51 (1.0)	1 (0.6)	>0.999
Pulmonary embolism, *n* (%)	5 (0.1)	0 (0.0)	>0.999
Pseudotumor cerebri, *n* (%)	20 (0.4)	1 (0.6)	0.523
Psychiatric disease, *n* (%)	67 (1.4)	2 (1.1)	>0.999
Depression, *n* (%)	490 (10.0)	19 (10.8)	0.701
Previous abdominal operation, *n* (%)	229 (4.7)	8 (4.5)	>0.999

BMI, body mass index. * Patients with complications had at least one of the following: bleeding, anastomosis leak, bowel obstruction, respiratory complications (including pneumonia), urinary infection, intraoperative complications, superficial infection, abdominal infection, small bowel perforation, acute renal failure, reoperation, readmission, conversion to open surgery, and death. ^†^ Some patients underwent more than one additional procedure during their OAGB.

**Table 6:** Univariate analysis of factors related to early postoperative complications after revisional OAGB performed from 2017–2021 (*n* = 1634).

Factor	Patients without Complications (*n* = 1552)	Patients with Complications * (*n* = 82)	*p*-Value
Female, *n* (%)	1172 (75.5)	59 (72.0)	0.511
Age (years), mean ± SD	43.7 ± 10.3	46.7 ± 10.0	0.018
Age ≥ 60 years, *n* (%)	87 (5.6)	7 (8.5)	0.324
BMI (kg/m^2^), mean ± SD	41.2 ± 4.8	41.4 ± 4.6	0.621
Baseline BMI (kg/m^2^) ≥ 50, *n* (%)	63 (4.9)	2 (3.6)	>0.999
Smoker, *n* (%)	126 (8.1)	9 (11.0)	0.406
Operative length (minutes), mean ± SD	81.0 ± 32.9	89.9 ± 50.9	0.809
Operative length ≥ 3 h, *n* (%)	28 (1.9)	7 (9.9)	<0.001
Additional concomitant procedures (%yes) ^†^, *n* (%)	1552 (73.9)	67 (81.7)	0.121
Lap. removal of gastric band, *n* (%)	894 (57.6)	51 (62.2)	0.425
Lap. cholecystectomy, *n* (%)	207 (13.3)	13 (15.9)	0.507
Lap. hiatal hernia repair, *n* (%)	221 (14.2)	13 (15.9)	0.630
Lap. partial gastrectomy, *n* (%)	88 (5.7)	11 (13.4)	0.014
Lap. ventral hernia repair, *n* (%)	26 (1.7)	4 (4.9)	0.060
Previous bariatric procedure			
Lap. adjustable band, *n* (%)	1118 (72.0)	64 (78.0)	0.257
Lap. sleeve gastrectomy, *n* (%)	473 (30.5)	22 (26.8)	0.539
Vertical banded gastroplasty, *n* (%)	74 (4.8)	9 (11.0)	0.033
Associated medical problems			
Hypertension, *n* (%)	390 (25.1)	20 (24.4)	>0.999
Diabetes mellitus, *n* (%)	419 (27.0)	27 (32.9)	0.253
Hyperlipidemia, *n* (%)	565 (36.4)	32 (39.0)	0.639
Obstructive Sleep Apnea, *n* (%)	113 (7.3)	8 (9.8)	0.385
Fatty liver disease, *n* (%)	988 (63.7)	52 (63.4)	>0.999
Aspirin treatment, *n* (%)	69 (4.4)	3 (3.7)	>0.999
Hypercoagulopathy, *n* (%)	13 (0.8)	1 (1.2)	0.515
Pulmonary embolism, *n* (%)	3 (0.2)	0 (0.0)	>0.999
Pseudotumor cerebri, *n* (%)	6 (0.4)	0 (0.0)	>0.999
Psychiatric disease, *n* (%)	19 (1.2)	1 (1.2)	>0.999
Depression, *n* (%)	196 (12.6)	9 (11.0)	0.864
Previous abdominal operation, *n* (%)	146 (9.4)	11 (13.4)	0.246

BMI, body mass index. * Patients with complications had at least one of the following: bleeding, anastomosis leak, bowel obstruction, death, respiratory complications (including pneumonia), urinary tract infection, intraoperative complications, superficial infection, intra-abdominal infection, small bowel perforation, acute renal failure, reoperation, readmission, and conversion to open surgery. ^†^ Additional procedures during the surgery—there could be more than one additional procedure in one surgery.

## Data Availability

Data from this study are available upon request.

## Supplementary Materials

The following supporting information can be downloaded at: https://www.mdpi.com/article/10.3390/jcm12216872/s1, Figure S1: Percentages of total pOAGB and rOAGB patients with complications within CDC categories.
