# Supplementary figures and images for: One Anastomosis Gastric Bypass in 6722 Patients: Early Outcomes from a Private Hospital Registry
Source: J Clin Med. 2023 Oct 31;12(21):6872. doi: 10.3390/jcm12216872 (PMC10648472; doi:10.3390/jcm12216872)

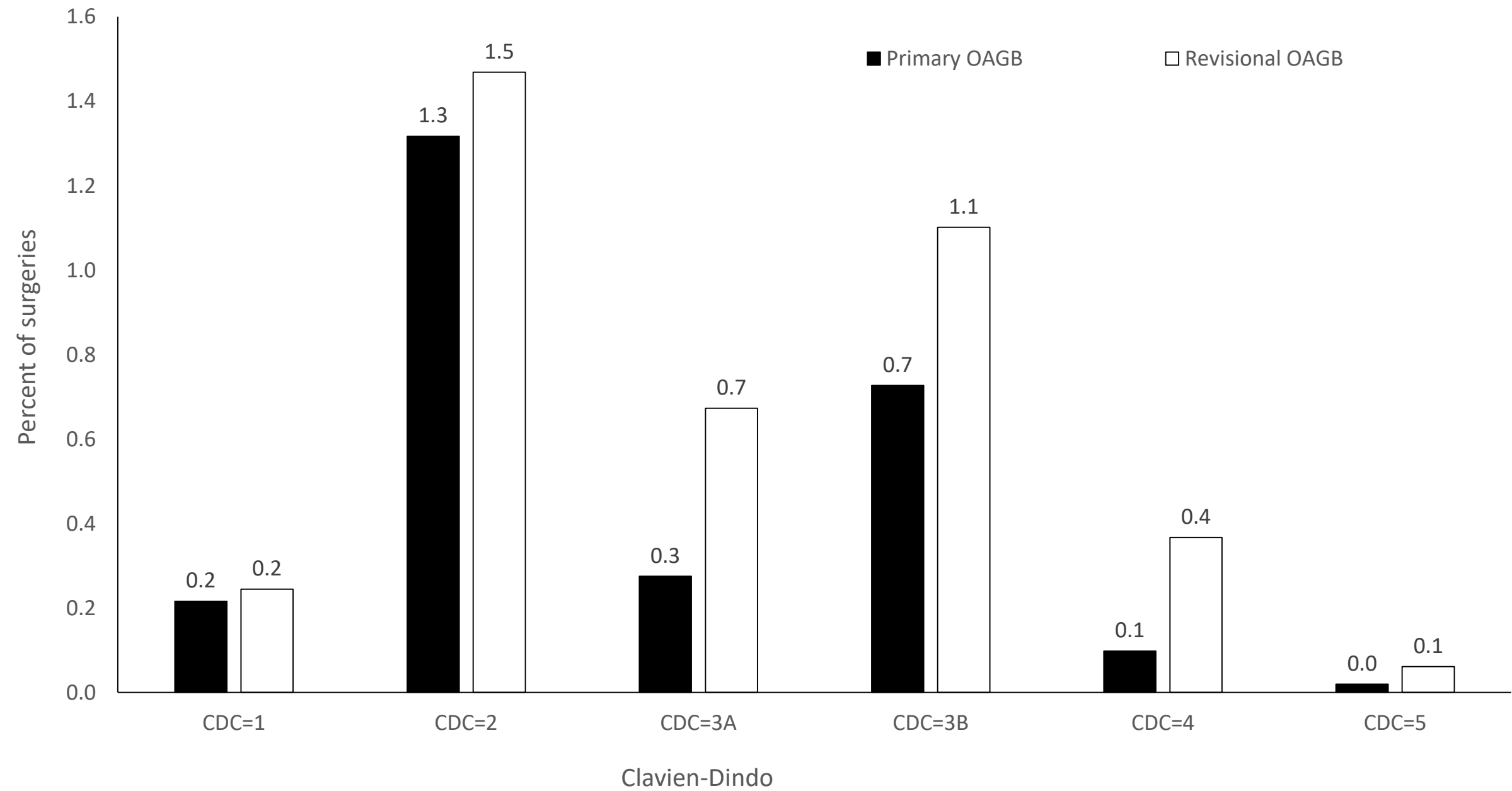

Supplement: Supplementary file 1 [file jcm-12-06872-s001.zip › jcm-2567171-supplementary.pdf]
